# The Influence of Parental Perfectionism and Parenting Styles on Child Perfectionism

**DOI:** 10.3390/children8090777

**Published:** 2021-09-04

**Authors:** Cláudia Carmo, Diana Oliveira, Marta Brás, Luís Faísca

**Affiliations:** 1 Psychology Research Centre (CIP), Universidade do Algarve, Campus de Gambelas, 8005-139 Faro, Portugal; dianafernandesoliveira@gmail.com (D.O.); mbras@ualg.pt (M.B.); 2 Centre for Biomedical Research (CBMR), Universidade do Algarve, Campus de Gambelas, 8005-139 Faro, Portugal; lfaisca@ualg.pt

**Keywords:** child perfectionism, parental perfectionism, adaptive and maladaptive perfectionism, parenting styles

## Abstract

Perfectionism is a significant transdiagnostic process related to the development and maintenance of several psychological disorders. The main models of the development of perfectionism focus on early childhood experiences and postulate that parental relation is an important factor for understanding this construct in children. The purpose of this study was to examine the relationship between child and parental perfectionism, seeking to evaluate the empirical support of the Social Learning Model and the Social Expectations Model and children’s perception of parenting styles. The present study included 119 children (51.2% girls, *M_age_* = 11.67 years) and their parents. Data were collected through administration of several self-report measures. The results show a relationship between the majority of the same parent and child perfectionism dimensions, thus providing supportive evidence for the Social Learning Model. Concerning the analysis of the role of gender in the transmission of perfectionism, observed fathers’ perfectionism only relates with the sons’ perfectionism, and mothers’ perfectionism relates with daughters’ perfectionism. Our findings allow for a deeper understanding of the role of the perception of an authoritarian parenting style in the development of maladaptive perfectionism. Mother and fathers’ perceived parenting styles contribute more to daughter than son perfectionism. The results contribute to expanding the understanding of the role of parental factors in the development of perfectionism.

## 1. Introduction

Perfectionism is a complex, transdiagnostic, multidimensional personality construct that encompasses both adaptive and maladaptive facets. One of the most popular and commonly used multidimensional conceptualizations of perfectionism was proposed by Hewitt and Flett [1], consisting in a tripartite model which includes both intrapersonal and interpersonal dimensions: self-oriented perfectionism (SOP; demanding perfection of oneself), socially prescribed perfectionism (SPP; perceiving others as demanding perfection of oneself) and other-oriented perfectionism (OOP; demanding perfection of others). 

The adverse consequences of perfectionism in the lives of youth (children and adolescents) are now widely recognized, including the impact on mental health and general well-being [2,3,4,5].

There is an increasing interest in studying perfectionism in children and adolescents [6,7,8] given that its features can be observed prior to adulthood and perfectionistic characteristics are considered to have roots in childhood.

In the literature on perfectionism, there is a variety of theoretical models aiming to explain how perfectionism may develop and all models highlight that childhood and adolescence are key periods for the development of this personality trait and that parents play a major role. Perfectionism develops over time and may be formed in the family of origin of the child, often resulting from parent–child interactions. 

The Social Expectations Model [9] proposes that perfectionism develops as a consequence of contingent parental approval combined with parental expectations and criticism. This means that parents teach their children that perfection and success are crucial to please them and to obtain their love and affection, whereas failure is not acceptable. Children whose parents have high performance expectations and criticize them when those expectations are not met are more prone to developing perfectionism by internalizing these expectations as well as the associated negative self-evaluation. 

Another model is the Social Learning Model [9], which focuses on the children’s tendency to model perfectionistic behaviors exhibited by their parents. This model suggests that children develop perfectionistic traits by observing and imitating their parents’ perfectionism, which may occur due to the frequent exposure to parental perfectionistic beliefs and behaviors or to the attempt to be as perfect as them. 

The literature reveals supporting evidence for both the social expectations [10] and social learning mechanisms of perfectionism development [11,12], with a few studies that have simultaneously tested these two models, suggesting that different pathways may lead to the development of different perfectionism dimensions [13,14].

Flett and colleagues [9] highlight that, in the study of the intergenerational transmission of perfectionism, gender may influence the outcome, emerging two concurrent hypotheses: the same-sex caregiver hypothesis and the primary-caregiver hypothesis. The first suggests that children tend to acquire features from the same-sex parent, meaning that sons will develop perfectionism through the interaction with their fathers, while daughters develop perfectionism through the interaction with their mothers. The latter theorizes that mothers are mainly responsible for the development of their children’s perfectionism, due to the longer period of time they spend with their children. This means children are more exposed to their mothers’ personality features and parenting styles than to their fathers’. Both hypotheses have received some support from research [11,14,15,16,17], creating a lack of consensus regarding the moderator effect of gender in the transmission of perfectionism.

In addition to parent perfectionism, research also suggests that there are other important factors regarding how parents influence the development of perfectionism in their children, namely, attachment styles [18], parental expectations [19], parental control [20] and, especially, parenting styles [21].

Baumrind [22] defined three parenting styles that primary caregivers use to interact with their children: authoritarian parenting, permissive parenting and authoritative parenting. Authoritarian parenting refers to parents that are rigorous, have excessively high expectations on how their children should conform to all imposed rules, attempting to shape, control and evaluate their children’s behaviors and attitudes. Permissive parents are warm and sensitive, highly accepting, make few demands and allow their children to regulate their own activities. Authoritative parents are affectionate and responsive to their children’s needs but are also capable of establishing rules and guide their children’s behaviors, finding a balance between affection and the rules establishment. 

Several studies [10,16,21,23,24,25,26,27,28] support the idea that perfectionism develops more easily in families with extremely critical parents and that an authoritarian parenting style may lead children to adopt a perfectionist orientation during the course of their lives. However, it is still not clear whether parenting styles are directly linked to the development of adaptive or maladaptive perfectionism facets.

While there is evidence showing a positive association between authoritarian parenting and maladaptive perfectionism [25,29,30], some studies reveal that this parenting style predicts both perfectionism facets [21,31,32,33].

Although progress has been made regarding the empirical support for the role of parents in the development of adolescents and children’s adaptive and maladaptive perfectionism, research is still relatively scarce and inclusive. This highlights the importance to continue to study the developmental trajectories of perfectionism, in order to better understand the origins of this personality trait.

The purpose of this study was to examine the relationship between child and parental perfectionism, seeking to evaluate the empirical support of the Social Learning Model and the Social Expectations Model [9]. The contribution of children’s perception of parenting styles on perfectionism development was also examined. We also sought to ascertain if the effect of these parental variables is homogeneous or whether it depends on the gender of both the parent and the child. 

## 2. Materials and Methods

### 2.1. Design

This is a quantitative study, with a cross-sectional and descriptive-correlational design.

### 2.2. Participants

Children from two public middle Portuguese schools were recruited for this study. A total of 268 questionnaires were sent to their parents, but only 154 questionnaires were returned, resulting in an overall response rate of 57.5%. After applying inclusion criteria (complete questionnaires, with responses both from mother and father), a final sample of 119 complete triads (mother, father, and child) was obtained. Children (58 boys and 61 girls) were aged between 10 and 14 years old (*M* = 11.67; *SD* = 1.18), 34 coming from the 5th grade, 44 from the 6th grade, 27 from the 7th grade and 14 from the 8th grade. Most of the children (82.4%) indicated that they lived with both parents. Mothers were aged between 29 to 55 years (*M* = 41.20; *SD* = 5.12) and fathers were aged between 31 to 68 years (*M* = 43.92; *SD* = 6.65). Concerning educational level, both parents attained college (39% and 27%, respectively, for mother and father), secondary school (28% and 25%, respectively) or middle school (19% and 24%).

### 2.3. Measures

#### 2.3.1. Sociodemographic Information

Sociodemographic data were collected using a Sociodemographic Questionnaire (SDQ). This instrument assesses participants’ personal, social, and demographic information. 

#### 2.3.2. Child Perfectionism

Child perfectionism was measured using the Child and Adolescent Perfectionism Scale (CAPS [34]; Portuguese version [35]). The CAPS is a 22-item self-report questionnaire that measures two aspects of perfectionism: Self-Oriented Perfectionism (SOP; 12 items) and Socially Prescribed Perfectionism (SPP; 10 items). Children are asked to rate each statement on a 5-point scale (1 = false—not at all true for me; 5 = very true for me). CAPS total score ranges from 22 to 110, with higher scores indicating greater perfectionism. The Portuguese version of CAPS shows adequate psychometric properties [35]: internal consistency and temporal stability indexes for the SOP scale (Cronbach´s α = 0.83; test-retest *r* = 0.69) and for the SPP (α = 0.85; *r* = 0.59) are good and quite similar to those obtained with the original version [34].

#### 2.3.3. Parent Perfectionism

Parent perfectionism was measured using the Hewitt and Flett’s Multidimensional Perfectionism Scale (HMPS [1]; Portuguese version [36]). The HMPS is a 45-item self-report scale that measures Self-Oriented Perfectionism (SOP; 15 items), Socially Prescribed Perfectionism (SPP; 15 items) and Other Oriented Perfectionism (OOP; 15 items). Participants respond to items on a 7-point scale (1 = strongly disagree; 7 = strongly agree). HMPS scores range from 45 to 315, with higher scores indicating greater perfectionism. The HMPS scales are internally consistent (SOP α = 0.89; SPP α = 0.86; OOP α = 0.79) and test–retest reliabilities are good (SOP *r* = 0.88; SPP *r* = 0.75; OOP *r* = 0.85). The Portuguese version of HMPS also presents adequate psychometric properties, showing good internal consistency (α = 0.89) and temporal stability (*r* = 0.85) for the total score.

#### 2.3.4. Parenting Styles

Parenting styles were measured using the Parental Authority Questionnaire (PAQ [37]; Portuguese version [38]). The PAQ is a 30-item instrument that measures three parenting styles: authoritative (10 items), authoritarian (10 items) and permissive (10 items) parenting styles, for both the mother and the father. Participants rate their level of agreement on a 5-point scale (1 = strongly disagree; 5 = strongly agree). On each PAQ subscale, scores range from 10 to 50, with higher scores indicating higher levels on each parental authority type. The Portuguese version of PAQ was translated and adapted by Morgado and colleagues [38] and has shown an adequate internal consistency (Authoritarian parenting style α = 0.77; Authoritative parenting style α = 0.78; Permissive parenting style α = 0.66).

### 2.4. Data Collection

The study was approved by the Portuguese Ministry of Education and the Directors of the Executive Boards of each selected school gave their authorization to collect data at their schools. The research team has then reunited with school administrators to schedule data collecting sessions, according to the availability of the teachers from the 5th to 8th grade. 

Since participants were minors, previous written consent was obtained from parents. All children also gave their assent to participate and completed the assessment protocol collectively in the classroom (Sociodemographic Questionnaire, Child and Adolescent Perfectionism Scale, and Parental Authority Questionnaire); whenever necessary, questions were clarified individually, while participants filled out the forms. Each child then received an envelope with an explaining note about the research and two HMPS questionnaires (one for the mother and one for the father) to deliver to their parents. Children brought from home the filled questionnaires in the closed envelope and handed them to their teachers, who in turn returned them to the researchers.

### 2.5. Data Analysis

Data analysis was carried out using IBM SPSS (version 25.0). *t*-tests were used to evaluate the significance of mean differences between groups and Cohen’s *d* to express the magnitude of those differences. Correlational analysis was based on the Pearson correlation coefficient *r*. Multiple regression models were used to evaluate the conjoint contribution of parental variables to explain children perfectionist dimensions. Significance level was set to 0.05; however, marginal significance (*p* < 0.1) was also reported.

## 3. Results

### 3.1. The Intergenerational Transmission of Perfectionism

The results obtained through children’s self-evaluation of perfectionism (Table 1) show that gender differences were negligible, although girls demonstrated a slightly higher level of SOP, an adaptive perfectionism dimension (Cohen’s *d* = 0.32, *p* = 0.086).

Parents’ self-reported levels of perfectionism (Table 2) were higher for fathers compared to mothers in all dimensions; however, differences were small and non-significant (Cohens’ *d* ≤ 0.2, *p* > 0.1).

In order to verify possible associations between parents and children’s perfectionistic tendencies, correlations were computed for the perfectionism scores, considering children’s gender. The results show only some statistically significant correlation coefficients (Table 3). Mother SOP levels were related to daughters’ SOP (*r* = 0.39); no statistically significant correlations were found between fathers’ SOP and children’s SOP. Fathers’ SOP was positively correlated with their sons’ SPP (*r* = 0.31). Mothers’ SPP was positively correlated with their daughters’ SPP (*r* = 0.33) and SOP (*r* = 0.25; here, the correlation is only marginally significant, *p* = 0.054). Parents’ OOP scores did not correlate with children’s perfectionism subscales. Significant correlations with total scores reflect the correlations already described at subscale level. 

Overall, since correlations were higher when the progenitor and child have the same gender, the results seem somehow to reflect the same-sex intergenerational transmission of perfectionism. However, correlations have moderate to weak magnitude (0.25 ≤ *r* ≤ 0.39). 

Given that parents’ SOP and SPP seem to contribute to the same perfectionism dimensions in children, a multiple regression model was used to evaluate the joint contribution of parents’ perfectionism dimensions to the development of child perfectionism traits (Table 4). Considering the coefficients of determination (*R^2^*) reported in Table 4, it can observe that mothers’ total contribution is only significant on daughters’ SOP (*R^2^* = 0.158) and SPP (*R^2^* = 0.130), while fathers’ total contribution is exclusively significant on sons’ SPP (*R^2^* = 0.163).

A detailed analysis of the contribution of individual variables (β standardized regression coefficients) indicates which parental perfectionism dimensions have a larger contribution to the development of child perfectionism. Fathers’ SOP and OOP are the perfectionism dimensions that most contribute to the development of sons’ SPP. Mothers’ SOP contributes to the development of daughters’ SOP, while mothers’ SPP contributes to daughters’ SPP.

### 3.2. The Influence of Perceived Parenting Styles on the Development of Perfectionism

Correlations between perceived parenting styles and perfectionism dimensions, according to child gender, were also explored (Table 5). The analysis of the results shows that the evaluation of an authoritarian parenting style is positively associated with all perfectionism dimensions, being particularly intense when it is done by daughters rather than sons. The stronger association observed in girls, compared to boys, is more evident for SOP. Concerning the association between perceived authoritative parenting and child perfectionism dimensions, correlations are non-significant (except for a marginally significant positive correlation between the daughter SOP dimension and their perception of mothers as having an authoritative parenting style, *r* = 0.25, *p* = 0.058). Perceived permissive parenting style have significant correlations exclusively with the SPP dimension, independently of the gender of the parent or of the child. In relation to CAPS Total, correlations reflect the association pattern described above.

In order to estimate the contribution of parenting styles to the explanation of adaptive and maladaptive perfectionism, multiple regression analysis was performed considering parenting styles as predictive variables and both perfectionism dimensions as dependent variables (Table 6).

Overall, the perceived parenting styles contribute more strongly to girls than boys levels of perfectionism. Maladaptive forms of perfectionism (SPP) seem to be more dependent on parenting styles; fathers’ parenting styles do not even significantly contribute to the SOP levels of their sons. Mother variables propitiate a somehow greater influence in perfectionism dimensions for both sexes. However, the contribution of each parenting style to perfectionism is similar for mother and father: authoritarian style is the major predictor of perfectionism, both for SOP and SPP. Finally, a more complex pattern of influence contributes to differences in girls’ SPP: while authoritarian and permissive parenting styles (both from mother and father) seem to contribute positively to this dimension of perfectionism, authoritative parenting style reduces the presence of such a maladaptive form of perfectionism among girls.

## 4. Discussion

The literature on the intergenerational transmission of child perfectionism is still inconclusive on the contribution of perfectionist dimensions and different parenting styles in the development of perfectionism in daughters and sons. In order to contribute to the understanding about the development of perfectionism during childhood, this cross-sectional study proposed to analyze the association of parental perfectionism and perceived parenting styles with the levels of child perfectionism.

Relatively to the levels of child perfectionism observed in the present sample, CAPS total scores were somehow higher than those obtained in the CAPS adaptation study for the Portuguese population [35], possibly due to the different characteristics of the samples of each study. The sample presented in the Portuguese adaptation study [35] was constituted by children aged from 11 to 18 years old (*M* = 15.8), while in the present study participants were aged between 10 and 14 years (*M* = 11.74). No statistically significant differences between genders for CAPS scores were found on both studies.

Parents’ self-evaluated perfectionism levels were coincident with the results of the Portuguese adaptation of HMPS [36], where SOP has shown the highest values, followed by SPP and OOP. As observed in this Portuguese adaptation study [36], no statistically significant gender differences were found for the various perfectionism dimensions.

The main purpose of this study was to explore the transmission of perfectionism from parents to children, by analyzing the contribution of parental perfectionism and parenting styles to the levels of perfectionism reported by their children. 

According to Social Learning Model [9], children may imitate parents’ perfectionism behaviors through a modeling process. The results of this study partially support the hypothesis that child perfectionism develops through the imitation of parents’ perfectionistic tendencies, since it was possible to observe that mothers’ SOP and SPP were significantly related to girls’ SOP and SPP, respectively. This result is also supported by most of the studies reported in the literature [2,11,14,15,16], suggesting that mothers that perceive others as holding them to high standards and expectations are more likely to have children who perceive others as being excessively demanding and holding unreasonable expectations.

The Social Expectations Model [9] emphasize that the intergenerational transmission of perfectionism may occur from the impact parents’ OOP has on children’s SPP. The present study refutes this idea: parents’ OPP did not related with children’s SPP. These results are similar to results found by Vieth and Trull [14], who also did not observe a relationship between these perfectionism dimensions. 

The analysis of the association between parental and children’s perfectionism, according to gender, allowed us to explore the intergenerational patterns of the transmission of perfectionism.

A positive and significant relationship was observed between mothers’ perfectionism (SOP and SPP) and the corresponding dimensions of daughters’ perfectionism. In turn, this was not true for the association between mother and son perfectionism. However, the fathers’ SOP and SPP dimensions do not correlate with the corresponding sons’ perfectionist dimensions. In the larger context of the Social Learning Model, these results seem to support both the hypothesis of the same-sex caregiver and the hypothesis of the primary caregiver, but only with respect to girls’ perfectionism. This exclusive positive relationship between the specific perfectionism dimensions of mothers and their daughters calls into question whether there is a double form of transmission from mother to daughter, offering support to both the main caregiver hypothesis (mother) and the same-sex caregiver hypothesis (female). The absence of a reliable association of the same perfectionism dimensions between fathers and mothers and their sons suggests that social learning, whether through same-sex caregiver modeling or through the primary caregiver modeling, may not be the relevant mechanism of the intergenerational transmission of perfectionism for boys.

However, a significant moderate association was observed between fathers’ SOP and sons’ SPP, suggesting that when fathers establish excessively high and unrealistic standards for themselves, sons tend to perceive that the significant others have rigid and excessively high expectations for themselves too. This pattern of influence seems to be specific to boys, since mothers’ SOP only correlates with daughters’ SOP. Furthermore, regression analysis detected an additional negative contribution of the fathers’ OPP dimension to the sons’ SPP, attenuating the role of parental expectations in this transmission mechanism. Further studies are needed to clarify if whether this same-sex mechanism of intergenerational transmission can be framed by the Social Learning Model and whether it is specific to male children.

Overall, the results of the correlational and regression analysis suggest that daughters’ perfectionism seems to be exclusively related to mothers’ perfectionism, although the modeling mechanism (same sex versus primary caregiver) could not be disentangled in this study; concerning boys’ perfectionism, it only relates to the father (but this relationship is exclusive between the fathers’ SOP and sons’ SPP dimensions). 

These results are partially corroborated by the study of Cook and Kearney [2], where mothers’ SPP was the only predictor of the development of children’s SOP and SPP, since the authors did not specify the role of gender in their study. 

Concerning the association between perceived parenting styles and child perfectionism, the results seem to point out that perception of an authoritarian parenting style is related to both perfectionism dimensions (SOP and SPP) in children, independently of gender. 

These findings corroborate results from other studies [21,31,32,33]. However, it is for the maladaptive perfectionism dimension (SPP) that the values are more evident, especially for boys. Indeed, while an authoritarian parenting style correlates moderately with both girls’ perfectionism dimensions (*r* > 0.4), for boys, correlations with SPP are similar (*r* > 0.4) but weaker for the SOP dimension (*r* < 0.3).

Kawamura et al. [26] assert that children that perceive their parents as being authoritarian, by internalizing parental criticism, may consequently develop harsh self-criticism. Perfectionistic children focus excessively on evaluative concerns, which may lead them to interpret any slight reprehension as a harsh critic directed to them. This relationship might happen because perfectionistic children are constantly focused on their own mistakes and more susceptible to remember situations in which their parents were critical about their performance. Damian et al. [10] also verified that adolescents who perceived their parents as having excessively high standards for themselves have increased their SPP levels. These results seem to corroborate the hypothesis that perfectionism is related to controlling and highly critical parenting styles, where parents are described as being demanding and imposing extremely elevated standards of performance on their children [25,26]. This association has also been reported in adulthood: a recent CBT Intervention study with young adults [30] reported that a perceived negative parenting style is associated with specific, maladaptive aspects of perfectionism.

Independently of child gender, perceived maternal parenting styles frequently reveal a somehow higher correlation than perceived paternal parenting styles. These results are consistent with the literature, in which the mother appears as the predominant figure, even though both mother and fathers’ harshness is associated with daughters’ perfectionism [16].

Considering gender, it was observed that perceived parental authoritarian was related to boy´s and girl´s perfectionism (SOP and SPP). These results are partially supported by Flett and colleagues´s study [24], where male students’ SPP correlated positively with both mother and fathers’ authoritarian parenting style; similar results were obtained in a study by Hibbard and Walton [25], which suggested that an authoritarian parenting style is positively associated to maladaptive perfectionism aspects. 

Concerning the association between perceived authoritative parenting and child perfectionism dimensions, correlations are non-significant (except for a marginally significant positive correlation between the daughter SOP dimension and their perception of mothers as having an authoritative parenting style).

In turn, perceived permissive parenting style was related exclusively with the SPP dimension, independently of the gender of the parent or of the child. Few studies have examined the relationship between permissive parenting style and perfectionism, however, their conclusions about this style is similar [25].

Regression analysis indicates that perceived parenting styles contribute more to daughter than son perfectionism. Maladaptive forms of perfectionism were particularly dependent on parenting styles; fathers’ parenting styles do not even significantly contribute to their the SOP levels reported by their sons. Mothers’ parenting styles propitiate a somehow greater influence in perfectionism dimensions for both sexes. However, the contribution of each parenting style to perfectionism is similar for mother and father: an authoritarian style is the major predictor of perfectionism, both for SOP and SPP. Finally, a more complex pattern of influence contributes to differences in girls’ SPP: while authoritarian and permissive parenting styles (both from mother and father) seem to contribute positively to this dimension of perfectionism, an authoritative parenting style seems to have a protective role against the presence of such a maladaptive form of perfectionism among girls.

The results obtained in this investigation seem to confirm the findings of other studies that have verified an association between parents and children’s perfectionism and the contribution of an authoritarian parenting style on maladaptive perfectionism in children. However, we cannot forget that the development of perfectionism involves other factors, such as sociocultural environment, peer and significant other (e.g., teachers, coaches) interactions as well as factors inherent to the child (e.g., temperament).

Some limitations should be noted, particularly of a methodological nature, namely, the use of a cross-sectional design and the use of self-evaluation and hetero-evaluation measures, which may be contaminated with social desirability. Aware of the difficulties inherent in a study of this nature, we suggest in the future the use of behavioral observations and a longitudinal design, allowing for a more effective understanding of the etiology and transmission of perfectionism from parent to child.

Finally, we also point out as a limitation the fact that the parental predictors of perfectionism are not exhausted in those we analyzed in this study. Studying all the parental variables hypothesized in the various explanatory models of perfectionism could help to understand the etiology of this personality trait. However, the results of the present study allowed us to reinforce the importance of the two parental factors in the understanding of perfectionism. Despite the limitations pointed out, this study provided empirical evidence for the relevance of fathers’ and mothers’ influence in promoting the positive and adaptive aspects, as well as the negative and maladaptive aspects, of perfectionism. 

## Figures and Tables

**Table 1:** Children perfectionism levels: differences according to gender.

CAPS	Boy (*n* = 58)	Girl (*n* = 61)			
	*M* (*SD*)	*M* (*SD*)	Cohen’s *d*	*t*	*p*
SOP (max. 60)	37.48 (7.80)	40.03 (8.23)	−0.32	−1.73	0.086
SPP (max. 50)	30.86 (7.97)	30.07 (7.78)	0.10	0.55	0.582
Total (max. 110)	68.34 (14.00)	70.10 (13.92)	−0.13	−0.68	0.495

Note. *M* = Mean; *SD* = Standard Deviation; *t* = Independent samples *t*-test; CAPS = Child and Adolescent Perfectionism Scale; SOP = Self-Oriented Perfectionism; SPP = Socially Prescribed Perfectionism; Total = Total perfectionism score assessed by CAPS.

**Table 2:** Perfectionism dimensions according to parent gender.

HMPS	Father (*n* = 119)	Mother (*n* = 119)			
	*M* (*SD*)	*M* (*SD*)	Cohen’s *d*	*t*	*p*
SOP (max. 105)	91.77 (15.47)	89.45 (16.66)	0.14	1.25	0.214
SPP (max. 105)	49.04 (11.75)	46.97 (11.43)	0.18	1.58	0.118
OOP (max. 105)	44.13 (7.48)	42.82 (8.54)	0.16	1.60	0.113
Total (max. 315)	199.70 (27.86)	194.53 (30.43)	0.18	1.59	0.115

Note. *M* = Mean; *SD* = Standard Deviation; *t* = Paired samples *t*-test (father and mother scores were paired); HMPS = Hewitt and Flett’s Multidimensional Perfectionism Scale; SOP = Self-Oriented Perfectionism; SPP = Socially Prescribed Perfectionism; OOP = Other Oriented Perfectionism; Total = Total perfectionism score assessed by HMPS.

**Table 3:** Pearson correlation between parents’ perfectionism dimensions (HMPS) and children’s perfectionism dimensions (CAPS), according to gender (boys: *n* = 58; girls: *n* = 61).

	CAPS–SOP		CAPS–SPP		CAPS–Total	
	Boy	Girl	Boy	Girl	Boy	Girl
HMPS–SOP						
Father	0.02	0.05	0.31 **	0.09	0.19	0.08
Mother	0.04	0.39 ***	0.20	0.16	0.14	0.32 **
HMPS–SPP						
Father	0.02	0.10	0.14	0.18	0.09	0.16
Mother	0.12	0.25 *	0.15	0.33 ***	0.15	0.33 ***
HMPS–OOP						
Father	−0.13	−0.06	−0.17	−0.18	−0.17	−0.13
Mother	−0.03	0.19	0.12	−0.12	0.06	0.05
HMPS–Total						
Father	−0.04	0.05	0.15	0.02	0.02	0.03
Mother	0.06	0.36 ***	0.24 *	0.19	0.15	0.32 **

Note. HMPS = Hewitt and Flett’s Multidimensional Perfectionism Scale; SOP = Self-Oriented Perfectionism; SPP = Socially Prescribed Perfectionism; OOP = Other Oriented Perfectionism; Total = total perfectionism score assessed by HMPS; CAPS = Child and Adolescent Perfectionism Scale; Total = Total perfectionism score assessed by CAPS. * *p* < 0.1; ** *p* < 0.05; *** *p* < 0.01.

**Table 4:** Total contribution of father and mother perfectionism dimensions (HMPS) in the development of son and daughter perfectionism (CAPS) (boys: *n* = 58; girls: *n* = 61).

	CAPS–SOP		CAPS–SPP	
	Boy	Girl	Boy	Girl
Father’s contribution	*R*^2^ = 0.019	*R*^2^ = 0.015	*R*^2^ = 0.163 **	*R*^2^ = 0.080
HMPS–SOP	β = 0.07	β = 0.05	β = 0.40 ***	β = 0.13
HMPS–SPP	β = −0.03	β = 0.08	β = −0.06	β = 0.14
HMPS–OOP	β = −0.15	β = −0.09	β = −0.28 **	β = −0.25 *
Mother’s contribution	*R^2^* = 0.015	*R^2^* = 0.158 **	*R^2^* = 0.050	*R^2^* = 0.130 **
HMPS–SOP	β = 0.04	β = 0.35 **	β = 0.16	β = 0.10
HMPS–SPP	β = 0.10	β = 0.07	β = 0.10	β = 0.29 *
HMPS–OOP	β = −0.05	β = 0.02	β = 0.03	β = −0.18

Note. β = Standardized regression coefficient; *R*^2^ = Coefficient of determination; SOP = Self-Oriented Perfectionism; SPP = Socially Prescribed Perfectionism; OOP = Other Oriented Perfectionism. * *p* < 0.1; ** *p* < 0.05; *** *p* < 0.01.

**Table 5:** Pearson correlation between perceived parenting styles and child perfectionism, according to child gender (boys: *n* = 58; girls: *n* = 61).

	CAPS–SOP		CAPS–SPP		CAPS–Total	
PAQ Subscales	Boy	Girl	Boy	Girl	Boy	Girl
Authoritarian						
Father	0.24 *	0.51 ****	0.39 ***	0.44 ****	0.35 ***	0.55 ****
Mother	0.28 **	0.47 ****	0.47 ****	0.52 ****	0.42 ****	0.57 ****
Authoritative						
Father	0.20	0.17	0.13	−0.19	0.19	−0.01
Mother	0.16	0.25 *	0.16	−0.17	0.18	0.05
Permissive						
Father	0.04	−0.13	0.31 **	0.33 ***	0.20	0.26 **
Mother	0.04	0.15	0.38 ***	0.37 ***	0.24 *	0.29 **

Note. CAPS = Child and Adolescent Perfectionism Scale (Flett et al., 2000); SOP = Self-Oriented Perfectionism; SPP = Socially Prescribed Perfectionism; CAPS total = Total perfectionism score assessed by CAPS; PAQ = Parental Authority Questionnaire. * *p* < 0.1; ** *p* < 0.05; *** *p* < 0.01; **** *p* < 0.001.

**Table 6:** Contribution of parenting styles to the development of child perfectionism (boys: *n* = 58; girls: *n* = 61).

	CAPS–SOP		CAPS–SPP	
PAQ Subscales	Boys	Girls	Boys	Girls
Paternal contribution	*R*^2^ = 0.082	*R*^2^ = 0.258 ****	*R*^2^ = 0.165 **	*R*^2^ = 0.300 ****
Authoritarian	0.26	0.48 ****	0.32 **	0.40 ****
Authoritative	0.13	0.10	−0.04	−0.26 **
Permissive	−0.13	0.00	0.15	0.24 **
Maternal contribution	*R^2^* = 0.110 *	*R^2^* = 0.234 ****	*R^2^* = 0.236 ***	*R^2^* = 0.405 ****
Authoritarian	0.41 **	0.44 ****	0.42 **	0.47 ****
Authoritative	0.03	0.16	−0.08	−0.33 ***
Permissive	−0.23	−0.04	0.13	0.27 **

Note. β = Standardized regression coefficient; *R*^2^ = Coefficient of determination; SOP = Self-Oriented Perfectionism; SPP = Socially Prescribed Perfectionism; PAQ = Parental Authority Questionnaire. * *p* < 0.1; ** *p* < 0.05; *** *p* < 0.01; **** *p* < 0.001.

## Data Availability

The database is available upon request to the first author.

## References

[1] Hewitt P.L., Flett G.L. (1991). Perfectionism in the Self and Social Contexts: Conceptualization, Assessment, and Association with Psychopathology. J. Pers. Soc. Psychol..

[2] Cook L.C., Kearney C.A. (2014). Parent Perfectionism and Psychopathology Symptoms and Child Perfectionism. Pers. Individ. Differ..

[3] Morris L., Lomax C. (2014). Review: Assessment, Development, and Treatment of Childhood Perfectionism: A Systematic Review. Child Adolesc. Ment. Health.

[4] Limburg K., Watson H.J., Hagger M.S., Egan S.J. (2017). The Relationship between Perfectionism and Psychopathology: A Meta-Analysis. J. Clin. Psychol..

[5] Karayağız Ş., Aktan T., Karayağız L.Z. (2020). Parental Attachment Patterns in Mothers of Children with Anxiety Disorder. Children.

[6] Flett G.L., Hewitt P.L., Besser A., Su C., Vaillancourt T., Boucher D., Munro Y., Davidson L.A., Gale O. (2016). The Child–Adolescent Perfectionism Scale: Development, Psychometric Properties, and Associations with Stress, Distress, and Psychiatric Symptoms. J. Psychoeduc. Assess..

[7] Harvey B.C., Moore A.M., Koestner R. (2017). Distinguishing Self-Oriented Perfectionism-Striving and Self-Oriented Perfectionism-Critical in School-Aged Children: Divergent Patterns of Perceived Parenting, Personal Affect and School Performance. Pers. Individ. Differ..

[8] Vicent M., Rubio-Aparicio M., Sánchez-Meca J., Gonzálvez C. (2019). A Reliability Generalization Meta-Analysis of the Child and Adolescent Perfectionism Scale. J. Affect. Disord..

[9] Flett G.L., Hewitt P.L., Oliver J.M., Macdonald S., Flett G.L., Hewitt P.L. (2002). Perfectionism in Children and Their Parents: A Developmental Analysis. Perfectionism: Theory, Research, and Treatment.

[10] Damian L.E., Stoeber J., Negru O., Bǎban A. (2013). On the Development of Perfectionism in Adolescence: Perceived Parental Expectations Predict Longitudinal Increases in Socially Prescribed Perfectionism. Pers. Individ. Differ..

[11] Appleton P.R., Hall H.K., Hill A.P. (2010). Family Patterns of Perfectionism: An Examination of Elite Junior Athletes and Their Parents. Psychol. Sport Exerc..

[12] Speirs Neumeister K.L., Williams K.K., Cross T.L. (2009). Gifted High-School Students’ Perspectives on the Development of Perfectionism. Roeper Rev..

[13] Speirs Neumeister K.L. (2004). Factors Influencing the Development of Perfectionism in Gifted College Students. Gift. Child Q..

[14] Vieth M., Trull T.J. (1999). Family Patterns of Perfectionism: An Examination of College Students and Their Parents. J. Pers. Assess..

[15] Cook L.C., Kearney C.A. (2009). Parent and Youth Perfectionism and Internalizing Psychopathology. Pers. Individ. Differ..

[16] Frost R.O., Lahart C.M., Rosenblate R. (1991). The Development of Perfectionism: A Study of Daughters and Their Parents. Cognit. Ther. Res..

[17] Yang H., Chen J. (2016). Learning Perfectionism and Learning Burnout in a Primary School Student Sample: A Test of a Learning-Stress Mediation Model. J. Child Fam. Stud..

[18] Ko A., Hewitt P.L., Cox D., Flett G.L., Chen C. (2019). Adverse Parenting and Perfectionism: A Test of the Mediating Effects of Attachment Anxiety, Attachment Avoidance, and Perceived Defectiveness. Pers. Individ. Differ..

[19] Curran T., Hill A.P., Madigan D.J., Stornæs A.V. (2020). A Test of Social Learning and Parent Socialization Perspectives on the Development of Perfectionism. Pers. Individ. Differ..

[20] Dieleman L.M., Soenens B., De Pauw S.S.W., Prinzie P., Vansteenkiste M., Luyten P. (2020). The Role of Parental Reflective Functioning in the Relation between Parents’ Self-Critical Perfectionism and Psychologically Controlling Parenting Towards Adolescents. Parenting.

[21] Walton G.E., Hibbard D.R., Coughlin C., Coyl-Shepherd D.D. (2020). Parenting, Personality, and Culture as Predictors of Perfectionism. Curr. Psychol..

[22] Baumrind D. (1966). Effects of Authoritative Control on Child Behavior. Child Dev..

[23] Domocus I.M., Damian L.E. (2018). The Role of Parents and Teachers in Changing Adolescents’ Perfectionism: A Short-Term Longitudinal Study. Pers. Individ. Differ..

[24] Flett G.L., Hewitt P.L., Singer A. (1995). Perfectionism and Parental Authority Styles. Individ. Psychol..

[25] Hibbard D.R., Walton G.E. (2014). Exploring the Development of Perfectionism: The Influence of Parenting Style and Gender. Soc. Behav. Pers..

[26] Kawamura K.Y., Frost R.O., Harmatz M.G. (2002). The Relationship of Perceived Parenting Styles to Perfectionism. Pers. Individ. Differ..

[27] Madjar N., Voltsis M., Weinstock M.P. (2015). The Roles of Perceived Parental Expectation and Criticism in Adolescents’ Multidimensional Perfectionism and Achievement Goals. Educ. Psychol..

[28] Rice K.G., Lopez F.G., Vergara D. (2005). Parental/Social Influences on Perfectionism and Adult Attachment Orientations. J. Soc. Clin. Psychol..

[29] Miller A.L., Lambert A.D., Neumeister K.L.S. (2012). Parenting Style, Perfectionism, and Creativity in High-Ability and High-Achieving Young Adults. J. Educ. Gift..

[30] Zikopoulou O., Nisyraiou A., Simos G. (2021). A Randomised Controlled CBT Intervention for Maladaptive Perfectionism : Outcome and Predictors. Psychiatry Int..

[31] Besharat M.A., Azizi K., Poursharifi H. (2011). The Relationship between Parenting Styles and Children’s Perfectionism in a Sample of Iranian Families. Procedia Soc. Behav. Sci..

[32] Craddock A.E., Church W., Sands A. (2009). Family of Origin Characteristics as Predictors of Perfectionism. Aust. J. Psychol..

[33] Gong X., Fletcher K.L., Bolin J.H. (2015). Dimensions of Perfectionism Mediate the Relationship between Parenting Styles and Coping. J. Couns. Dev..

[34] Flett G.L., Hewitt P.L., Boucher D.J., Davidson L.A., Munro Y. (2000). The Child—Adolescent Perfectionism Scale: Development, Validation and Association with Adjustment.

[35] Bento C., Pereira A.T., Maia B., Marques M., Soares M.J., Bos S., Valente J., Gomes A., Azevedo M.H.P., Macedo A. (2010). Perfectionism and Eating Behaviour in Portuguese Adolescents. Eur. Eat. Disord. Rev..

[36] Soares M., Gomes A., Macedo A., Azevedo M. (2003). Escala Multidimensional de Perfeccionismo: Adaptação à População Portuguesa. Rev. Port. Psicossomática.

[37] Buri J. (1991). Parental Authority Questionnaire. J. Pers. Assess..

[38] Morgado J., Maroco J., Miguel M., Machado R., Dias C. Estilos Parentais e Suporte Social Em Adolescentes: Relação Com o Comportamento Escolar. Proceedings of the VI Simpósio Nacional de Investigação em Psicologia.

